# Long-Term Outcomes of Induction Chemotherapy Followed by Chemo-Radiotherapy as Intensive Neoadjuvant Protocol in Patients with Esophageal Cancer

**DOI:** 10.3390/cancers12123614

**Published:** 2020-12-03

**Authors:** Nicola Simoni, Michele Pavarana, Renato Micera, Jacopo Weindelmayer, Valentina Mengardo, Gabriella Rossi, Daniela Cenzi, Anna Tomezzoli, Paola Del Bianco, Simone Giacopuzzi, Giovanni De Manzoni, Renzo Mazzarotto

**Affiliations:** 1 Department of Radiotherapy, Ospedale Civile Maggiore, Azienda Ospedaliera Universitaria Integrata Verona, 37126 Verona, Italy; renato.micera@aovr.veneto.it (R.M.); gabriella.rossi@aovr.veneto.it (G.R.); renzo.mazzarotto@aovr.veneto.it (R.M.); 2 Department of Oncology, Ospedale G.B. Rossi, Azienda Ospedaliera Universitaria Integrata Verona, 37126 Verona, Italy; michele.pavarana@aovr.veneto.it; 3 Department of General and Upper G.I. Surgery, Ospedale Civile Maggiore, Azienda Ospedaliera Universitaria Integrata Verona, 37126 Verona, Italy; jacopo.weindelmayer@aovr.veneto.it (J.W.); valentina.mengardo@gmail.com (V.M.); simone.giacopuzzi@univr.it (S.G.); giovanni.demanzoni@univr.it (G.D.M.); 4 Department of Radiology, Ospedale Civile Maggiore, Azienda Ospedaliera Universitaria Integrata Verona, 37126 Verona, Italy; daniela.cenzi@aovr.veneto.it; 5 Department of Pathology, Ospedale G.B. Rossi, Azienda Ospedaliera Universitaria Integrata Verona, 37126 Verona, Italy; anna.tomezzoli@aovr.veneto.it; 6 Clinical Research Unit, Istituto Oncologico Veneto IOV-IRCCS, 35100 Padova, Italy; paola.delbianco@iov.veneto.it

**Keywords:** induction chemotherapy, chemo-radiotherapy, neoadjuvant treatment, esophageal cancer

## Abstract

**Simple Summary:**

Neoadjuvant chemo-radiotherapy (nCRT) represents a standard approach for both Squamous Cell Carcinoma (SCC) and Adenocarcinoma (ADC) of the esophagus, leading to a 10–15% improvement in survival rate as compared with surgery alone in clinical trials. In this observational study, we report the efficacy and safety of an intensive nCRT protocol in the daily clinical practice, including 122 patients treated with induction chemotherapy, followed by concomitant chemo-radiotherapy, and surgery. Our findings showed good long-term survival and high pathological complete response (pCR) rates, with acceptable side-effects. Notably, the oncological outcome was the same in ADC and SCC responder patients. Although the nCRT protocol here reported represents a distinctive single-center experience, our results contribute to better define the role of an intensive neoadjuvant approach as a reliable therapy for the treatment of locally advanced esophageal cancer, and enrich the current literature on this challenging context.

**Abstract:**

Background: A phase II intensive neoadjuvant chemo-radiotherapy (nCRT) protocol for esophageal cancer (EC) was previously tested at our Center with promising results. We here present an observational study to evaluate the efficacy of the protocol also in “real life” patients. Methods: We retrospectively reviewed 122 ECs (45.1% squamous cell (SCC) and 54.9% adenocarcinoma (ADC)) treated with induction docetaxel, cisplatin, and 5-fluorouracil (TCF), followed by concomitant TCF and radiotherapy (50–50.4 Gy/25–28 fractions), between 2008 and 2017. Primary endpoints were overall survival (OS), event-free survival (EFS) and pathological complete response (pCR). Results: With a median follow-up of 62.1 months (95% CI 50–67.6 months), 5-year OS and EFS rates were 54.8% (95% CI 44.7–63.9) and 42.7% (95% CI 33.1–51.9), respectively. A pCR was observed in 71.1% of SCC and 37.1% of ADC patients (*p* = 0.001). At multivariate analysis, ypN+ was a significant prognostic factor for OS (Hazard Ratios (HR) 4.39 [95% CI 2.36–8.18]; *p* < 0.0001), while pCR was a strong predictor of EFS (HR 0.38 [95% CI 0.22–0.67]; *p* < 0.0001). Conclusions: The nCRT protocol achieved considerable long-term survival and pCR rates also in “real life” patients. Further research is necessary to evaluate this protocol in a watch-and-wait approach.

## 1. Introduction

Esophageal Cancer (EC) represents a major health problem worldwide, ranking seventh among leading causes of cancer-related death [1]. Multimodal treatment, including chemotherapy, radiotherapy, and surgery, is currently accepted as standard of care for locally advanced stage disease [2,3]. Several randomized trials demonstrated a survival benefit with neoadjuvant chemo-radiotherapy (nCRT) followed by surgery, compared to surgery alone, both in patients with Squamous Cell Carcinoma (SCC) and Adenocarcinoma (ADC) of the esophagus and gastroesophageal junction (EGJ) [4,5,6,7,8,9]. Notably, responders to nCRT have a better prognosis than non-responders [10], and an intensification of the preoperative approach is often advocated to improve oncological outcomes [11].

In our previous experience, an intensive nCRT protocol was tested in a phase II trial, with encouraging results [12]. The nCRT protocol schedule consisted of an induction phase of weekly administered docetaxel, cisplatin, and 5-fluorouracil (TCF) for 3 weeks, followed by concomitant TCF administered weekly for 5 weeks along with radiotherapy (50–50.4 Gy in 25–28 fractions). Remarkably, a pathological complete response (pCR) was obtained in 47% of patients with a 5-year overall survival (OS) rate of 43% (77% for pCR group). These results could be explained by the use of a more intensive chemotherapy schedule and an increased radiotherapy dose compared to other preoperative approaches reported in the literature [6]. Based on these results, this protocol was considered the standard nCRT for both advanced esophageal SCCs and ADCs treated in our center.

However, since trial participants do not represent the population as a whole, applying this protocol in the daily practice could have led to poorer results. [13]. Based on this consideration, we performed a novel analysis of the efficacy and safety of this nCRT protocol in the daily clinical practice.

## 2. Results

### 2.1. Baseline Characteristics

A total of 122 consecutive patients were included in the analysis: 55 (45.1%) with SCC and 67 (54.9%) with ADC. Baseline characteristics are outlined in Table 1.

### 2.2. Treatment Completion

One hundred and nineteen (97.5%) patients underwent concurrent chemo-radiotherapy after the first induction phase, while three (2.5%) were excluded: two due to disease progression during induction chemotherapy, and one due to acute intestinal occlusion requiring surgery. One hundred and sixteen (97.5%) patients received the full prescribed radiation dose, while 3 (2.5%) did not complete the treatment schedule due to toxicity. In five (4.2%) patients the prescription dose was reduced to 45 Gy due to patient’s frailty or to large field nodal volume. The median relative dose intensity (RDI) for the chemotherapy schedule was 0.86 (0.74–0.95). During the induction phase, no reduction in the administered chemotherapy doses was needed, and the average relative dose intensity (RDI) was 0.96 (0.88–1). Instead, during the concomitant phase, the average RDI was reduced to 0.77 (0.61–0.90), with a similar reduction for all drugs (average RDI 0.75 [0.57–0.88], 0.79 [0.60–0.91] and 0.75 [0.65–0.98] for docetaxel, cisplatin, and 5-fluorouracil, respectively). Table S1 (Supplementary Material) describes relative dose intensity (RDI), dose density, as well as nCRT protocol treatment details.

One hundred and seven (87.7%) patients underwent surgery. Radical resection (R0) was achieved in 105 patients (98.1% of resected patients). Table 2 reports details on surgery and pathological assessment.

### 2.3. Treatment Outcomes

**Figure 1:**
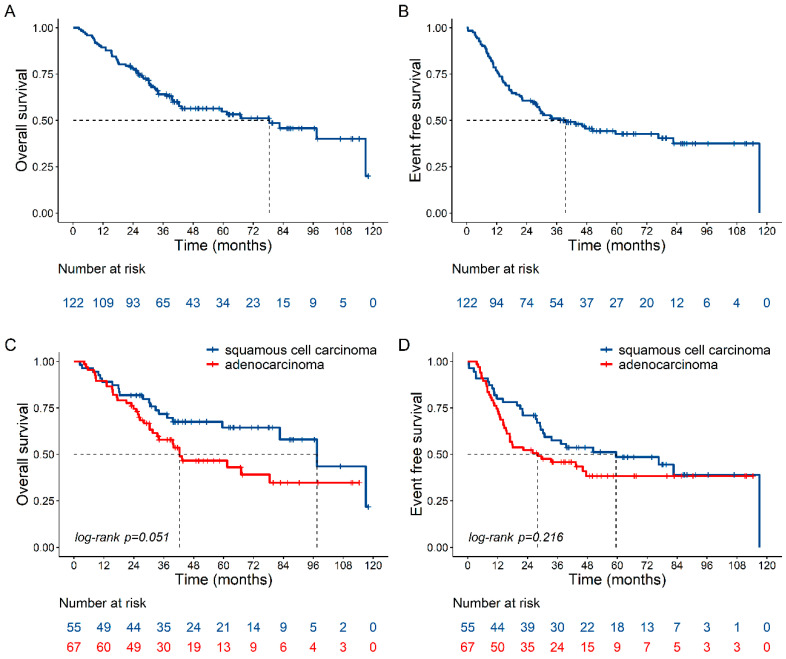
Overall Survival (OS) and Event-Free Survival (EFS) estimated by Kaplan–Meier method. (**A**) OS and (**B**) EFS of the entire cohort; (**C**) OS and (**D**) EFS as a function of histotype (squamous cell carcinoma vs. adenocarcinoma).

The estimated median follow-up time was 62.1 months (95% CI 49.0–67.6 months). Median OS and EFS of the entire cohort were 78.5 months (95% CI 42.3-NE [not estimable]) and 39.5 months (95% CI 27.3–82.6), respectively (Figure 1A,B), and increased in resected patients to 97.4 months (Hazard Ratios (HR) 0.24 95% CI 0.12–0.47, *p* < 0.0001) and 46.2 months (HR 0.28 95% CI 0.15–0.52, *p* < 0.0001), respectively. The OS rates at 1, 2, 3, and 5 years were 89.3% (95% CI 82.4–93.7), 77.8% (95% CI 69.4–84.2), 64.2% (95% CI 54.7–72.2), and 54.8% (95% CI 44.7–63.9), and the comparable EFS rates were 77.0% (95% CI 68.5–83.5), 60.7% (95% CI 51.4–68.7), 51.1% (95% CI 41.8–59.6), and 42.7% (95% CI 33.1–51.9), respectively. Median OS and EFS did not significantly differ between SCC versus ADC patients (Figure 1C,D).

### 2.4. Pathological Complete Response

**Figure 2:**
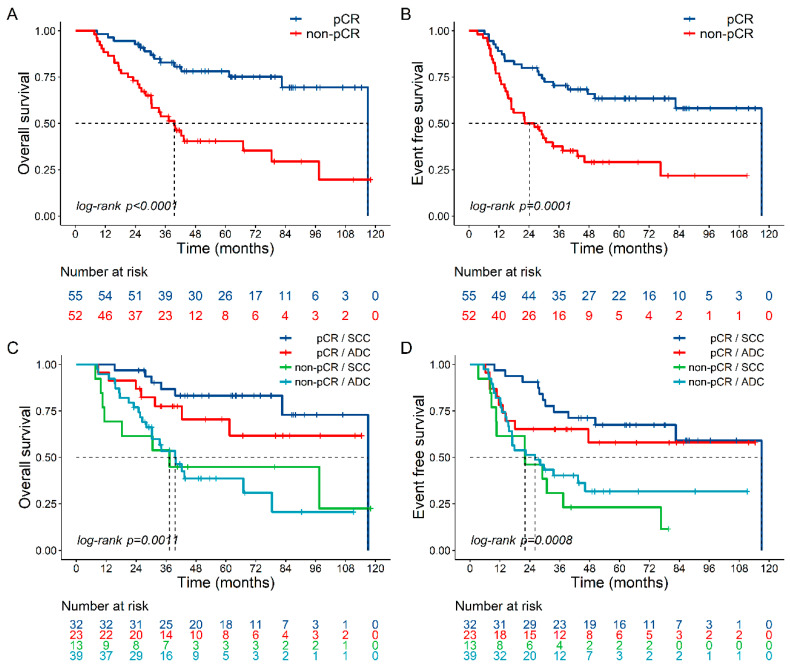
(**A**) Overall Survival (OS) and (**B**) Event-Free Survival (EFS) estimated by Kaplan–Meier method as a function of pathological complete response (pCR vs. non-pCR) in resected patients. (**C**) OS and (**D**) EFS as a function of pCR and histotype (squamous cell carcinoma vs. adenocarcinoma). pCR: pathological complete response; SCC: squamous cell carcinoma; ADC: adenocarcinoma.

Among resected patients, pCR was achieved in 51.4% (55/107) of patients, including 71.1% (32/45) of SCC and 37.1% (23/62) of ADC patients (*p* < 0.001). Median OS and EFS were particularly high in pCR cases, being 117 months (HR 0.30 95% CI 0.16–0.56, *p* < 0.0001) and 117 months (HR 0.35 95% CI 0.20–0.61, *p* < 0.0001), respectively (Figure 2A,B), with a similar trend for SCC and ADC patients (Figure 2C,D). The 3- and 5-year OS rates were 82.8% (95% CI 69.5–90.7) and 70.5% (95% CI 56.4–80.8), and the comparable EFS rates were 78.2% (95% CI 63.9–87.4) and 63.5% (95% CI 48.6–75.1), respectively, in pCR patients, as compared with 53.8% (95% CI 38.9–66.6) and 37.6% (95% CI 24.5–50.7), and 40.4% (95% CI 25.9–54.6) and 29.1% (95% CI 16.6–42.8) in non-pCR patients, respectively (*p* < 0.001). Tumor relapse occurred in 48 resected patients (44.9%), with a loco-regional pattern in 7 (6.5%) (Table S2, Supplementary Material).

In the univariate analysis, gender, pCR, pTstage, pNstage and Tumor Regression Grade (TRG) were significantly associated with OS and EFS (Table 3). In the multivariate analysis, pNstage remained a significant predictor for OS (the HR of pN1 cases with respect to pN0 cases was 4.39 (95% CI 2.36–8.18; *p* < 0.0001)), while pCR remained significant for EFS (the HR of pCR cases with respect to non-pCR cases was 0.38 (95% CI 0.22–0.67; *p* < 0.0001) (Table 3)).

### 2.5. Protocol Toxicity and Postoperative Complications

Of the 119 (97.5%) patients who completed the nCRT protocol, 92 (77.3%) experienced at least one adverse event. Details of toxic effects are shown in Table 4. Thirty-two (26.9%) patients had grade ≥3 acute hematological toxicity, while 23 (19.3%) had acute grade ≥3 non hematological events. Overall, a potentially treatment-related death occurred in 4 (3.4%) patients.

None of the patients who underwent surgery died within 30 days after resection or in-hospital. Fifty-nine (55.1%) patients had at least one post-operative complication (Table 5), most of which were mild [14]. Considering severe complications alone (Clavien Dindo ≥3b according to the Esophagectomy Complications Consensus Group [15]), 8 (7.3%) cases required reoperation or ICU. Of these, surgical serious events occurred in 5 (4.6%) patients while medical severe complications were reported in 3 (2.8%).

## 3. Discussion

Over the last 15 years, neoadjuvant chemo-radiotherapy and peri-operative chemotherapy have become the standard approaches for locally advanced EC, leading to a 10–15% improvement in long-term survival rates as compared with surgery alone in clinical trials [7,8,9,16]. However, which is the optimal strategy is still under debate. This observational study reports the efficacy and safety of an intensive nCRT protocol in the daily clinical practice for locally advanced EC. To the best of our knowledge, this study includes one of the largest cohorts of patients treated with induction chemotherapy, followed by chemo-radiotherapy, as the preoperative approach in EC. This nCRT protocol has previously been tested at our institution in a phase II trial, with good long-term survival (median OS 55 months) and pCR (47% of patients) [12]. The results of the present study, with an estimated median follow-up of 62.1 months, confirm the high OS and EFS rate (median 78.5 and 39.5 months, respectively) also in “real life” patients. This finding is relevant, and emphasizes the efficacy, in terms of survival benefit, for neoadjuvant chemo-radiotherapy when added to surgery in patients with EC.

Noteworthy, pCR was achieved in 51.4% of resected patients, one of the highest percentages reported so far [17]. Indeed, a pCR is considered one of the best available predictors of outcome for EC patients who undergo chemo-radiation therapy followed by esophagectomy [18,19]. A recent MDACC cohort study showed that pCR was associated with an improved survival (median OS 71.28 months for pCR versus 35.87 for non-pCR cases, *p* = 0.002) [10]. Of the 911 treated patients, 218 (23.9%) achieved a pCR, with a rate of 32.2% for SCC and of 23.1% for ADC (*p* = 0.06). In our study, pCR patients achieved a 5-year OS rate of 70.5% (versus 37.6% in non-pCR patients), with a similar survival trend for SCC and ADC responder patients (Figure 2C,D). This result supports the role of pCR as a trustworthy surrogate predictor marker of survival advantage.

In the ChemoRadiotherapy for Oesophageal cancer followed by Surgery Study (CROSS) trial, pCR rate was 29%, with a significantly larger number of SCC patients (49% versus 23% for ADC, *p* = 0.008) [7]. In our study, 32 of 45 (71.1%) SCC patients had a pCR in the surgical specimen. This percentage is remarkable and confirms the greater sensitivity of SCC to full-dose chemo-radiotherapy as previously reported by other authors [20]. Another issue is whether surgery on demand is advisable in selected clinical complete responder patients. In a subgroup analysis of our study, we found that the percentage of pCR was significantly higher for SCC vs. ADC tumors (17/21, 81% vs. 4/16, 25%, *p* = 0.002) in females, while no significant difference was observed in males (15/24, 63% for SCC vs. 19/46, 41% for ADC, *p* = 0.15). Moreover, in patients with a pCR, median OS and EFS were particularly high in females (not achievable versus 82.6 months in males, *p* = 0.01, and not achievable versus 33.6 months in males, *p* = 0.002, respectively). Based on these results, the female population with SCC seems to be the ideal candidate for a watch-and-wait approach. The ongoing randomized SANO trial, comparing salvage surgery with immediate surgery in clinical complete responders after nCRT, will provide results over the next few years [21,22].

Controversy exists over the optimal neoadjuvant approach for gastroesophageal junction (EGJ) adenocarcinomas [23]. Neoadjuvant chemo-radiation is associated with an increased local control of the tumor compared with perioperative chemotherapy alone, but this does not translate into an increased survival [24]. Furthermore, the pCR rates in ADCs treated with chemo-radiotherapy are significantly worse than SCCs, being less than 20–25% [6,7,20]. An increase in pCR rate, correlated with the use of higher doses of radiotherapy, compared to 41.4 Gy used in the CROSS trial [7], has been described in the literature. In detail, the use of doses between 45 and 50.4 Gy, in combination with carboplatin-paclitaxel, produced a pCR in 29–36% of treated patients, with acceptable toxicity [25,26,27]. In our series the pCR rate for ADCs was noticeably high, being 37.1%, and in this subset of patients both median OS and EFS were 117 months. This may be due to the use of an intensive schedule with docetaxel, cisplatin plus 5-fluorouracil (5-FU) during the induction phase and concomitant with radiotherapy (RT), as well as to the 50–50.4 Gy dose administered in this protocol that could have helped maximize local response. This finding further supports the potential effectiveness and generalizability of the use of nCRT in ADC of the esophagus, as a reliable or even better alternative to perioperative chemotherapy in selected patients, although the design of the study does not permit to draw definitive conclusions, due to the lack of a control group [28]. However, many ADCs are extremely resistant to chemo-radiotherapy: these ADC patients may not benefit from this treatment but are exposed to its negative consequences such as toxicity and delayed surgical therapy. To this regard, a multicenter, randomized phase II study on BIRC3-expression driven therapy (nCRT versus upfront surgery), in patients with resectable ADC of the esophagus and EGJ, is currently ongoing (BoRgES trial, NCT04269083) at the authors’ institution.

According to the literature [29], this study confirms that nodal downstaging (ypN0) is a strong predictor for OS. We can assume that nodal response might be as important as downstaging on the primary tumor, and that a poor nodal response cannot be compensated even by radical surgery, thus representing a reliable biological marker for poorer survival. Instead, pCR remains the predominant prognostic factor for EFS, presumably indicating that complete response to nCRT corresponds to a particularly favorable tumor biology or treatment efficacy or both. This latter finding is particularly intriguing for the squamous histology. As mentioned above, if the pCR rate is extremely high in this subgroup, and the consequent EFS markedly prolonged, close observation with salvage surgery might be an embraceable option to improve patients’ quality of life (QoL) [30].

One potential criticism regarding the use of this intensive nCRT protocol is toxicity, leading to death in about 3% of treated patients. Thus, its use should be recommended only in specialized centers. However, the vast majority of patients were able to complete the planned preoperative treatment, and, notably, the subsequent surgery was not jeopardized by the nCRT protocol. The R0 surgical rate is also remarkable, amounting to 98.1% of resected patients (86.1% for the entire cohort). Hence, considering that tumor shrinkage after nCRT can significantly increase the R0 resection rate that 73.8% of patients achieved a ypN0 and 51.4% a pCR, and that nCRT adverse events did not represent a contraindication for surgery, we can assert that the protocol survival benefit was not counteracted by an excessive toxicity.

Our study presents some limitations. Indeed, it is an observational study, with a 10-year enrollment period, during which some variations in diagnostic accuracy, management of patients and post-operative surveillance occurred. Moreover, the indication to the nCRT protocol was defined on the basis of our previous experience and as a distinctive practice of our multidisciplinary team, thus our results could be biased by the patient selection process. Finally, this analysis included different histologies (SCC and ADC), which could have added heterogeneity to the outcomes measured.

## 4. Materials and Methods

### 4.1. Study Design

This study is an Institutional Review Board (IRB)-approved (Number DBCES001) observational single-center analysis of prospectively collected data, designed to assess the real-life effectiveness and safety of our nCRT protocol in patients with SCC and ADC of the esophagus and gastroesophageal junction. We considered all consecutive patients treated at our Institution from January 2008 to December 2017. The following perioperative data were collected: baseline demographics, diagnostic work-up, neoadjuvant protocol details, intra-operative findings, and post-operative data. According to the main international guidelines [2], patients with Siewert III type tumors were treated as gastric cancers, while patients with SC cervical tumors were assigned to definitive chemo-radiotherapy and therefore excluded from the analysis.

### 4.2. Staging

The pre-treatment staging consisted of clinical examination, blood chemistries including tumor markers, contrast-enhanced total body CT scan, fluorodeoxyglucose positron-emission tomography (^18^FDG-PET/CT), esophagogastroduodenoscopy with biopsies, and endoscopic ultrasound (EUS). In SCC patients, tracheobronchoscopy, esophageal magnetic resonance (MR), and cervical ultrasound were also performed. Patients were staged according to the Union for International Cancer Control [UICC] TNM cancer staging [31] and the therapeutic approach was defined by the institutional multidisciplinary tumor board.

### 4.3. Chemo-Radiotherapy Schedule

Treatment schedule consisted of a first phase of induction chemotherapy for 3 weeks (days 1–22), followed by a second phase of concurrent chemotherapy and radiotherapy for 5 weeks (days 29–63), as previously described [12]. Briefly, the chemotherapy treatment plan was as follows: docetaxel 35 mg/m^2^ and cisplatin 25 mg/m^2^ on days 1, 8, 15, 29, 36, 43, 50 and 57 plus 5 fluorouracil (5-FU) 180 mg/m^2^ as protracted venous infusion (c.i.) on days 1 to 21 and 150 mg/m^2^ c.i. on days 29 to 63. The detailed treatment schedule is represented in Table S3 (Supplementary Material).

Radiation therapy (RT) was delivered concurrently with chemotherapy starting on day 29. The prescribed dose was 50–50.4 Gy delivered in 25–28 fractions. The gross tumor volume (GTV) included the primary lesion and any regionally involved lymph nodes. The GTV was contoured using data from CT scan, EUS, and PET/CT fusion scans. The clinical target volume (CTV) was generated by expanding the GTV tumor by 3 cm cranially and caudally and 1 cm radially, while positive lymph nodes were uniformly expanded by 1 cm. The CTV was usually completed with the addition of the elective nodal irradiation (ENI) volume [32]. A CTV-to-PTV margin of 8–10 mm was applied. Until 2013, RT was delivered using three-dimensional conformal radiotherapy (3D-CRT). From 2014, 3D-CRT was replaced by intensity-modulated radiotherapy (IMRT) and volumetric modulated arc radiotherapy (VMAT). Image-guided radiation therapy (IGRT) was routinely used.

### 4.4. Restaging, Surgery and Pathological Analysis

Patients were restaged with pretreatment work-up procedures between the fourth and fifth week after treatment completion. Response evaluation was performed using response evaluation criteria in solid tumors (RECIST and PERCIST Criteria) [33,34]. Surgery with radical intent was performed 6 to 8 weeks after nCRT completion. A Two- or 3-field lymph node dissection was performed based on tumor site and clinical nodal status at diagnosis. Abdominal D2 and standard mediastinal lymphadenectomy was the standard approach for ADC. Extension to the recurrent nerve chain nodes or a complete 3-field lymphadenectomy was performed for SCC based on node involvement. Peritoneal lavage cytology was evaluated in all ADC patients. Positive cytology was considered to be metastatic. Surgical complications were registered according to Clavien Dindo Classification [15]. A positive resection margin (R1) was defined as vital tumor cells within 1 mm of the proximal and distal resection margins, while a circumferential margin was considered involved if neoplastic cells were found at the cut margin. Pathological complete response (pCR) was defined as no vital tumor cells in the resection specimen (ypT0N0M0), and Tumor Regression Grade (TRG) was scored according to a modified Mandard score system [35].

### 4.5. Follow-Up

Follow-up examination was performed every 6 months after surgery for resected cases and every 3 months after nCRT protocol completion for non-resected patients. The follow-up schedule included: total body contrast-enhanced CT scan, esophagogastroscopy, tumor markers, neck endoscopic ultrasound in SCC and a clinical assessment. Toxicity data were collected during follow-up according to common terminology criteria for adverse events (CTCAE) version 4.0 [36].

### 4.6. Statistical Analysis

Quantitative variables were described as median and interquartile range (IQR) or mean and standard deviation (SD), categorical variables were summarized as counts and percentages. The median follow-up time was based on the reverse Kaplan-Meier estimator. Primary endpoints considered were OS, event-free survival (EFS) and pCR. Secondary endpoint was toxicity.

OS was the time from the start of induction chemotherapy to death, and EFS was calculated from the start of induction chemotherapy to the date of a documented disease progression, relapse, or death. Patients who did not develop an event during the study period were censored at the date of last observation. The survival probabilities were estimated using the Kaplan-Meier method and reported with their 95% confidence interval (CI). Comparisons among strata were performed using the log-rank test. Hazard ratios (HR) and 95% CI for each group were estimated using univariate Cox proportional hazards models. No deviation from the proportional hazards assumption were found by the numerical methods of Lin et al. [37]. The independent role of each covariate in predicting survival was verified in a multivariable model considering all characteristics significantly associated with the outcome in the univariate analyses. Associations were assessed using the χ2 or Fisher exact test as appropriate. All statistical tests were two-sided and a *p* value <0.05 was considered statistically significant. Statistical analyses were performed using the RStudio (RStudio: Integrated Development for R. RStudio Inc., Boston, MA, US).

## 5. Conclusions

In conclusion, this intensive neoadjuvant schedule with induction chemotherapy followed by chemo-radiotherapy, based on docetaxel, cisplatin, 5-fluorouracil, and 50–50.4 Gy radiotherapy, achieves considerable results in terms of survival and pCR rate also in “real life” patients, largely counterbalancing the risk of not negligible adverse events. Noteworthy, the protocol does not jeopardize the achievement of radical resection and does not increase the rate of postoperative complications. Further studies are necessary to evaluate the use of this protocol also in a watch-and-wait approach.

## Figures and Tables

**Table 1:** Clinical and tumor characteristics of 122 patients.

		*N*	%
**Age, years**	Median (IQR)	63 (57–79)	
	<60	42	34.4
	60–69	51	41.8
	≥70	29	23.8
**Gender**	Female	46	37.7
	Male	76	62.3
**Histology**	SCC	55	45.1
	ADC	67	54.9
**BMI**	Median (IQR)	22.9 (19.6–27.5)	
**ASA score** °	I	5	4.7
	II	75	70
	III	27	25.3
**Tumor location**	Upper third	18	14.8
	Middle third	28	22.9
	Distal third	36	29.5
	EGJ	40	32.8
**Tumor length, cm**	Median (IQR)	5.5 (4.1–7.0)	
**Clinical T stage**	1	2	1.6
	2	12	9.8
	3	98	80.3
	4	9	7.4
	X *	1	0.8
**Clinical N stage**	N0	19	15.6
	N+	103	84.4
**Clinical stage group**	IIA	17	13.9
	IIB	12	9.8
	III	92	75.5
	X ^§^	1	0.8

IQR: interquartile range; SCC: squamous cell carcinoma; ADC: adenocarcinoma; BMI: body mass index; EGJ: gastroesophageal junction; * clinical T stage not evaluable because of incomplete endoscopic ultrasound (EUS); ^§^ clinical stage TxN1; ° only patients who underwent surgery.

**Table 2:** Details on surgery and pathological assessment.

		*N*	%
Total		119 ^§^	
Surgery	No	12	10.1
	W&W *	5	41.7
	Death before surgery °	5	41.7
	PD during nCRT	1	8.3
	Patient unfit for surgery	1	8.3
	Yes	107	89.9
	McKeown ^+^	36	33.7
	Ivor-Lewis ^#^	65	60.7
	Total Gastrectomy ^	6	5.6
Months between nCRT	Median	1.97	
and surgery	(IQR)	(1.63–2.30)	
pT	0	63	58.9
	1	14	13.1
	2	14	13.1
	3	13	12.1
	4	3	2.8
pN	N0	79	73.8
	N1	19	17.8
	N2	5	4.7
	N3	4	3.7
pM	M0	105	98.1
	M1	2	1.9
Pathological complete response	T0N0M0	55	51.4
Radicality	R0	105	98.1
	R1	2	1.9
Positive Nodes	Median (IQR)	0 (0–1)	
Retrieved Nodes	Median (IQR)	26.5 (19–35)	
LN ratio	Median (IQR)	0 (0–0.03)	
TRG ^§^	1	58	54.2
	2	17	15.9
	3	6	5.6
	4	8	7.5

PD: progression disease; nCRT: neoadjuvant chemoradiotherapy; IQR: interquartile range; LN: lymph node; TRG: tumor regression grade. ^§^ Patients evaluated for surgery after chemo-radiotherapy; * watch-and-wait strategy following evidence of a complete response to nCRT protocol (bite-on-bite biopsies proven); ° 4 (4.1%) patients due to presumable nCRT toxicity and 1 (0.8%) patient for causes not tumor related; ^§^ information on TRG was missing in 19 patients (the sum of patients for this column does not match the total due to missing data); ^+^ McKeown procedure: Tri-incisional subtotal esophagectomy with cervical esophago-gastric anastomosis; ^#^ Ivor-Lewis procedure: partial esophagectomy with right intrathoracic esophago-gastrostomy; ^ total gastrectomy and distal esophagectomy with intramediastinal anastomosis.

**Table 3:** Univariate and Multivariate Hazard Ratios (HR) and 95% CIs of factors associated with OS and EFS in resected patients.

**OS**		**Univariable Analysis**		**Multivariable Analysis**	
**Variable**		**HR (95% CI)**	***p* Value**	**HR (95% CI)**	***p* Value**
Age	<60	1			
	60–69	0.91 (0.50–1.65)	0.7582		
	≥70	0.73 (0.35–1.53)	0.4066		
Gender	Male	1		1	
	Female	0.44 (0.24–0.83)	0.01	0.37 (0.16–0.84)	0.017
Histology	SCC	1			
	ADC	1.72 (0.99–2.99)	0.051		
pCR	No	1			
	Yes	0.30 (0.16–0.56)	<0.0001		
pT stage	T0	1			
	T1–4	2.06 (1.14–3.71)	0.0162		
pN stage	N0	1		1	
	N1	5.11 (2.76–9.47)	<0.0001	4.39 (2.36–8.18)	<0.0001
TRG	1	1			
	2–4	2.71 (1.29–5.67)	0.008		
**EFS**		**Univariable Analysis**		**Multivariable Analysis**	
**Variable**		**HR (95% CI)**	***p* Value**	**HR (95% CI)**	***p* Value**
Age	<60	1			
	60–69	0.8 (0.5–1.4)	0.4614		
	≥70	0.7 (0.4–1.3)	0.3014		
Gender	Male	1		1	
	Female	0.43 (0.25–0.75)	0.0029	0.44 (0.22–0.87)	0.0184
Histology	SCC	1			
	ADC	1.36 (0.84–2.20)	0.22		
pCR	No	1		1	
	Yes	0.35 (0.20–0.61)	<0.0001	0.38 (0.22–0.67)	<0.0001
pT stage	T0	1			
	T1–4	1.99 (1.17–3.38)	0.0109		
pN stage	N0	1			
	N1	3.73 (2.14–6.49)	<0.0001		
TRG	1	1			
	2–4	2.09 (1.09–3.99)	0.03		

OS: overall survival; EFS: event-free survival; HR: hazard ratio; CI: confidence interval; SCC: squamous cell carcinoma; ADC: adenocarcinoma; pCR: pathological complete response; TRG: tumor regression grade.

**Table 4:** Neoadjuvant chemoradiotherapy protocol-related toxicity.

At Least One Adverse Event	92/119 (77.3%)		
	Grade 1/2	Grade 3/4	Grade 5
Nausea, *n* (%)	57 (47.9)	6 (5.0)	0 (0.0)
Vomiting, *n* (%)	16 (13.4)	3 (2.5)	0 (0.0)
Esophagitis *, *n* (%)	42 (35.3)	12 (10.1)	0 (0.0)
Diarrhea, *n* (%)	30 (25.2)	0 (0.0)	0 (0.0)
Fatigue, *n* (%)	49 (41.2)	7 (5.9)	0 (0.0)
Skin toxicity, *n* (%)	15 (12.6)	2 ^°^ (1.7)	0 (0.0)
Neutropenia, *n* (%)	36 (30.3)	25 (21.0)	2 ^§^ (1.7)
Thrombocytopenia, *n* (%)	14 (11.8)	2 (1.7)	0 (0.0)
Anemia, *n* (%)	4 (3.4)	4 (3.4)	0 (0.0)
Cardiac toxicity, *n* (%)	0 (0.0)	0 (0.0)	2 ^^^ (1.7)
Radiation pneumonia, *n* (%)	0 (0.0)	1 (0.8)	0 (0.0)
Aorto-esophageal fistula, *n* (%)	0 (0.0)	1 ^ⁿ^ (0.8)	0 (0.0)

* appearance or worsening; ^§^ neutropenic fever and sepsis; ^°^ taxane-related skin reaction; ^^^ heart failure; ^ⁿ^ requiring an intravascular stent implantation.

**Table 5:** Postoperative complications.

Complications	Clavien Dindo Classification	Events,
		*n* (%)
Global, *n* patients (%)		59 (55.1)
	Grade I	8 (7.4)
	Grade II	22 (20.6)
	Grade III a	21 (19.6)
	Grade III b	5 (4.6)
	Grade IV a	3 (2.8)
	Grade IV b	0 (0.0)
	Grade V	0 (0.0)
Surgical, *n* events (%)		39 (36.4)
	Grade I	11 (10.3)
	Grade II	8 (7.5)
	Grade III a	15 (14.0)
	Grade III b	5 (4.6)
	Grade IV a	0 (0.0)
	Grade IV b	0 (0.0)
	Grade V	0 (0.0)
Medical, *n* events (%)		30 (28.0)
	Grade I	1 (0.9)
	Grade II	19 (17.8)
	Grade III a	7 (6.5)
	Grade III b	0 (0.0)
	Grade IV a	3 (2.8)
	Grade IV b	0 (0.0)
	Grade V	0 (0.0)
Frequent complication, *n* events (%)	Anastomotic leak	9 (8.4)
	Pulmonary/Pleuric Complication	21 (19.6)
	Cardiac Complication	17 (15.8)
Median LOS, days (range)		10 (6–41)
Mortality (30 days or In-Hospital)		0 (0.0)

LOS: Length of Hospital Stay.

## Supplementary Materials

The following are available online at https://www.mdpi.com/2072-6694/12/12/3614/s1. **Table S1.** Neoadjuvant chemo-radiotherapy protocol details. **Table S2.** Incidence and pattern of failure distribution among resected patients (first site of recurrence). **Table S3.** Neoadjuvant chemo-radiotherapy protocol schedule.

## References

[1] Siegel R., Ma J., Zou Z., Jemal A. (2014). Cancer statistics, 2014. CA Cancer J. Clin..

[2] NCCN (2020). NCCN Guidelines—Esophageal and Esophagogastric Junction Cancers. Version 1. https://www.nccn.org/professionals/physician_gls/pdf/esophageal.pdf.

[3] Lordick F., Mariette C., Haustermans K., Obermannová R., Arnold D., ESMO Guidelines Committee (2016). Oesophageal cancer: ESMO Clinical Practice Guidelines for diagnosis, treatment and follow-up. Ann. Oncol..

[4] Walsh T.N., Noonan N., Hollywood D., Kelly A., Keeling N., Hennessy T.P. (1996). A comparison of multimodal therapy and surgery for esophageal adenocarcinoma. N. Engl. J. Med..

[5] Bosset J.F., Gignoux M., Triboulet J.P., Tiret E., Mantion G., Elias D., Lozach P., Ollier J.C., Pavy J.J., Mercier M. (1997). Chemoradiotherapy followed by surgery compared with surgery alone in squamous-cell cancer of the esophagus. N. Engl. J. Med..

[6] Tepper J., Krasna M.J., Niedzwiecki D., Hollis D., Reed C.E., Goldberg R., Kiel K., Willett C., Sugarbaker D., Mayer R. (2008). Phase III trial of trimodality therapy with cisplatin, fluorouracil, radiotherapy, and surgery compared with surgery alone for esophageal cancer: CALGB 9781. J. Clin. Oncol..

[7] Van Hagen P., Hulshof M.C., van Lanschot J.J., Steyerberg E.W., van Berge Henegouwen M.I., Wijnhoven B.P., Richel D.J., Nieuwenhuijzen G.A.P., Hospers G.A.P., Bonenkamp J.J. (2012). Preoperative chemoradiotherapy for esophageal or junctional cancer. N. Engl. J. Med..

[8] Shapiro J., van Lanschot J.J.B., Hulshof M.C.C.M., van Hagen P., van Berge Henegouwen M.I., Wijnhoven B.P.L., van Laarhoven H.W.M., Nieuwenhuijzen G.A.P., Hospers G.A.P., Bonenkamp J.J. (2015). Neoadjuvant chemoradiotherapy plus surgery versus surgery alone for oesophageal or junctional cancer (CROSS): Long-Term results of a randomised controlled trial. Lancet Oncol..

[9] Yang H., Liu H., Chen Y., Zhu C., Fang W., Yu Z., Mao W., Xiang J., Han Y., Chen Z. (2018). Neoadjuvant chemoradiotherapy followed by surgery versus surgery alone for locally advanced squamous cell carcinoma of the esophagus (NEOCRTEC5010): A phase III multicenter, randomized, open-label clinical trial. J. Clin. Oncol..

[10] Blum Murphy M., Xiao L., Patel V.R., Maru D.M., Correa A.M., Amlashi F.G., Liao Z., Komaki R., Lin S.H., Skinner H.D. (2017). Pathological complete response in patients with esophageal cancer after the trimodality approach: The association with baseline variables and survival-The University of Texas MD Anderson Cancer Center experience. Cancer.

[11] Iams W.T., Villaflor V.M. (2017). Neoadjuvant treatment for locally invasive esophageal cancer. World J. Surg..

[12] Pasini F., de Manzoni G., Zanoni A., Grandinetti A., Capirci C., Pavarana M., Tomezzoli A., Rubello D., Cordiano C. (2013). Neoadjuvant therapy with weekly docetaxel and cisplatin, 5-fluorouracil continuous infusion, and concurrent radiotherapy in patients with locally advanced esophageal cancer produced a high percentage of long-lasting pathological complete response: A phase 2 study. Cancer.

[13] Zarbin M. (2019). Real life outcomes vs. clinical trial results. J. Ophthalmic Vis. Res..

[14] Low D.E., Alderson D., Cecconello I., Chang A.C., Darling G.E., D’Journo X.B., Griffin S.M., Hölscher A.H., Hofstetter W.L., Jobe B.A. (2015). International consensus on standardization of data collection for complications associated with esophagectomy: Esophagectomy Complications Consensus Group (ECCG). Ann. Surg..

[15] Clavien P.A., Barkun J., de Oliveira M.L., Vauthey J.N., Dindo D., Schulick R.D., de Santibañes E., Pekolj J., Slankamenac K., Bassi C. (2009). The Clavien-Dindo classification of surgical complications: Five-year experience. Ann. Surg..

[16] Ronellenfitsch U., Schwarzbach M., Hofheinz R., Kienle P., Kieser M., Slanger T.E., Jensen K., GE Adenocarcinoma Meta-analysis Group (2013). Perioperative chemo(radio)therapy versus primary surgery for resectable adenocarcinoma of the stomach, gastroesophageal junction, and lower esophagus. Cochrane Database Syst. Rev..

[17] De Gouw D.J.J.M., Klarenbeek B.R., Driessen M., Bouwense S.A.W., van Workum F., Fütterer J.J., Rovers M.M., Ten Broek R.P.G., Rosman C. (2019). Detecting pathological complete response in esophageal cancer after neoadjuvant therapy based on imaging techniques: A diagnostic systematic review and meta-analysis. J. Thorac. Oncol..

[18] Chirieac L.R., Swisher S.G., Ajani J.A., Komaki R.R., Correa A.M., Morris J.S., Roth J.A., Rashid A., Hamilton S.R., Wu T.T. (2005). Posttherapy pathologic stage predicts survival in patients with esophageal carcinoma receiving preoperative chemoradiation. Cancer.

[19] Meredith K.L., Weber J.M., Turaga K.K., Siegel E.M., McLoughlin J., Hoffe S., Marcovalerio M., Shah N., Kelley S., Karl R. (2010). Pathologic response after neoadjuvant therapy is the major determinant of survival in patients with esophageal cancer. Ann. Surg. Oncol..

[20] Bollschweiler E., Metzger R., Drebber U., Baldus S., Vallböhmer D., Kocher M., Hölscher A.H. (2009). Histological type of esophageal cancer might affect response to neo-adjuvant radiochemotherapy and subsequent prognosis. Ann. Oncol..

[21] Noordman B.J., Spaander M.C.W., Valkema R., Wijnhoven B.P.L., van Berge Henegouwen M.I., Shapiro J., Biermann K., van der Gaast A., van Hillegersberg R., Hulshof M.C.C.M. (2018). Detection of residual disease after neoadjuvant chemoradiotherapy for oesophageal cancer (preSANO): A prospective multicentre, diagnostic cohort study. Lancet Oncol..

[22] Noordman B.J., Wijnhoven B.P.L., Lagarde S.M., Boonstra J.J., Coene P.P.L.O., Dekker J.W.T., Doukas M., van der Gaast A., Heisterkamp J., Kouwenhoven E.A. (2018). Neoadjuvant chemoradiotherapy plus surgery versus active surveillance for oesophageal cancer: A stepped-wedge cluster randomised trial. BMC Cancer.

[23] Petrelli F., Ghidini M., Barni S., Sgroi G., Passalacqua R., Tomasello G. (2019). Neoadjuvant chemoradiotherapy or chemotherapy for gastroesophageal junction adenocarcinoma: A systematic review and meta-analysis. Gastric Cancer.

[24] Al-Batran S.E., Homann N., Pauligk C., Goetze T.O., Meiler J., Kasper S., Kopp H.G., Mayer F., Haag G.M., Luley K. (2019). Perioperative chemotherapy with fluorouracil plus leucovorin, oxaliplatin, and docetaxel versus fluorouracil or capecitabine plus cisplatin and epirubicin for locally advanced, resectable gastric or gastro-oesophageal junction adenocarcinoma (FLOT4): A randomised, phase 2/3 trial. Lancet.

[25] Mukherjee S., Hurt C.N., Gwynne S., Sebag-Montefiore D., Radhakrishna G., Gollins S., Hawkins M., Grabsch H.I., Jones G., Falk S. (2017). NEOSCOPE: A randomised phase II study of induction chemotherapy followed by oxaliplatin/capecitabine or carboplatin/paclitaxel based pre-operative chemoradiation for resectable oesophageal adenocarcinoma. Eur. J. Cancer.

[26] Nabavizadeh N., Shukla R., Elliott D.A., Mitin T., Vaccaro G.M., Dolan J.P., Maggiore R.J., Schipper P.H., Hunter J.G., Thomas C.R. (2016). Preoperative carboplatin and paclitaxel-based chemoradiotherapy for esophageal carcinoma: Results of a modified CROSS regimen utilizing radiation doses greater than 41.4 Gy. Dis. Esophagus.

[27] Paireder M., Jomrich G., Kristo I., Asari R., Rieder E., Beer A., Ilhan-Mutlu A., Preusser M., Schmid R., Schoppmann S.F. (2020). Modification of preoperative radiochemotherapy for esophageal cancer (CROSS protocol) is safe and efficient with no impact on surgical morbidity. Strahlenther. Onkol..

[28] Zhou H.Y., Zheng S.P., Li A.L., Gao Q.L., Ou Q.Y., Chen Y.J., Wu S.T., Lin D.G., Liu S.B., Huang L.Y. (2020). Clinical evidence for association of neoadjuvant chemotherapy or chemoradiotherapy with efficacy and safety in patients with resectable esophageal carcinoma (NewEC study). EClinicalMedicine.

[29] Zanoni A. (2017). Nodal downstaging in esophageal and esophagogastric junction cancer: More important than ever. J. Thorac. Dis..

[30] Park S.R., Yoon D.H., Kim J.H., Kim Y.H., Kim H.R., Lee H.J., Jung H.Y., Lee G.H., Song H.J., Kim D.H. (2019). A randomized phase III trial on the role of esophagectomy in complete responders to Preoperative chemoradiotherapy for esophageal Squamous cell Carcinoma (ESOPRESSO). Anticancer Res..

[31] Sobin L.H., Gospodarowicz M.K., Wittekind C. (2009). TNM Classification of Malignant Tumors.

[32] Wu A.J., Bosch W.R., Chang D.T., Hong T.S., Jabbour S.K., Kleinberg L.R., Mamon H.J., Thomas C.R., Goodman K.A. (2015). Expert consensus contouring guidelines for intensity modulated radiation therapy in esophageal and gastroesophageal junction cancer. Int. J. Radiat. Oncol. Biol. Phys..

[33] Eisenhauer E.A., Therasse P., Bogaerts J., Schwartz L.H., Sargent D., Ford R., Dancey J., Arbuck S., Gwyther S., Mooney M. (2009). New response evaluation criteria in solid tumours: Revised RECIST guideline (version 1.1). Eur. J. Cancer.

[34] Wahl R.L., Jacene H., Kasamon Y., Lodge M.A. (2009). From RECIST to PERCIST: Evolving Considerations for PET response criteria in solid tumors. J. Nucl. Med..

[35] Mandard A.M., Dalibard F., Mandard J.C., Marnay J., Henry-Amar M., Petiot J.F., Roussel A., Jacob J.H., Segol P., Samama G. (1994). Pathologic assessment of tumor regression after preoperative chemoradiotherapy of esophageal carcinoma. Clinicopathologic correlations. Cancer.

[36] National Cancer Institute (2010). Common Terminology Criteria for Adverse Events (CTCAE) v.4.03.

[37] Lin D.Y., Wei L.J., Ying Z. (1993). Checking the cox model with cumulative sums of martingale-based residuals. Biometrika.

