# Supplementary material for: Long-Term Outcomes of Induction Chemotherapy Followed by Chemo-Radiotherapy as Intensive Neoadjuvant Protocol in Patients with Esophageal Cancer
Source: Cancers (Basel). 2020 Dec 3;12(12):3614. doi: 10.3390/cancers12123614 (PMC7761709; doi:10.3390/cancers12123614)
Supplement: Supplementary file 1 [file cancers-12-03614-s001.pdf]

cancers

Article

Long-Term Outcomes of Induction Chemotherapy Followed by Chemo-Radiotherapy as Intensive Neoadjuvant Protocol in Patients with Esophageal Cancer

Nicola Simoni [1,*,†], Michele Pavarana [2,†], Renato Micera [1], Jacopo Weindelmayer [3], Valentina Mengardo [3], Gabriella Rossi [1], Daniela Cenzi [4], Anna Tomezzoli [5], Paola Del Bianco [6], Simone Giacopuzzi [3], Giovanni De Manzoni [3,‡] and Renzo Mazzarotto [1,‡]

Table S1. Neoadjuvant chemo-radiotherapy protocol details.

**Average RDI Protocol**	**Median (IQR)**	**0.86 (0.74–0.95)**	
**Average RDI induction**	Median (IQR)	0.96 (0.88–1)	
- **Docetaxel**		0.96 (0.88–0.96)	
- **Cisplatin**		0.99 (0.89–1)	
- **5-Fluorouracil**		0.98 (0.84–1)	
**Duration of induction (days)**	Median (IQR)	21 (21–21)	
**Average RDI concomitant**	Median (IQR)	0.77 (0.61–0.90)	
- **Docetaxel**		0.75 (0.57–0.88)	
- **Cisplatin**		0.79 (0.60–0.91)	
- **5-Fluorouracil**		0.79 (0.65–0.98)	
**Duration of concomitant (days)**	Median (IQR)	35 (34–36)	
**Radiotherapy dose**	Median (IQR)	50 (50–50.4)	
	50–50.4 Gy	111	93.3%
	< 50 Gy	8	6.7%
**Technique**	3D-CRT	74	62.2%
	IMRT/VMAT	45	37.8%

RDI: relative dose intensity; IQR: interquartile range; Gy: gray; 3D-CRT: three-dimensional conformal radiotherapy; IMRT: intensity-modulated radiotherapy; VMAT: volumetric modulated arc therapy; nCRT: neoadjuvant chemoradiotherapy.

Table S2. Incidence and pattern of failure distribution among resected patients (first site of recurrence).

**Total, n (%)**	**48/107 (44.9%)**

**Liver, n (%)**	**8 (16.7)**
**Lung, n (%)**	**5 (10.4)**
**Extra-regional nodes, n (%)**	**3 (6.3)**
**Brain, n (%)**	**10 (20.8)**
**Bone, n (%)**	**2 (4.2)**
**Others, n (%)**	**1 (2.1)**

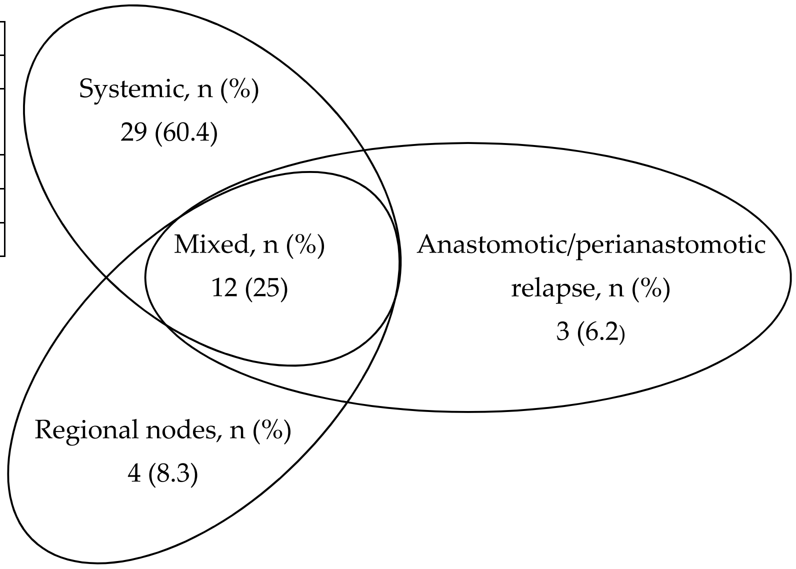

	**pCR**	**non-pCR**	
Total, n (%)	17/55 (30.9)	31/52 (59.6)	***p* = 0.003**
Systemic, n (%)	12 (70.6)	17 (54.8)	
Loco-regional, n (%)	2 (11.8)	5 (16.1)	*p* = 0.29
Mixed, n (%)	3 (17.6)	9 (29.1)	

	**SCC**	**ADC**	
Total, n (%)	18/45 (40.0)	30/62 (48.4)	*p* = 0.39
Systemic, n (%)	6 (33.3)	22 (73.3)	
Loco-regional, n (%)	4 (22.2)	3 (10.0)	***p* = 0.006**
Mixed, n (%)	8 (44.5)	5 (16.7)	

SCC: squamous cell carcinoma; ADC: adenocarcinoma; pCR: pathological complete response.

Table S3. Neoadjuvant chemo-radiotherapy protocol schedule.

		**Induction Chemotherapy (First Phase)**				**Concurrent Chemo-radiotherapy (Second Phase)**					
**Drug**	**mg/m²**	**Days**									
		**1**	**8**	**15**	22	29	36	43	50	57	
**T**	35	↓	↓	↓		↓	↓	↓	↓	↓	
**C**	25	↓	↓	↓		↓	↓	↓	↓	↓	
**F**	c.i.	180 × 21 days				150 × 35 days					
**RT§**						|||||	|||||	|||||	|||||	|||||	|||

c.i.: continuous infusion; T: docetaxel; C: cisplatin; F: 5 fluorouracil; RT: radiotherapy. Doses of 5-FU are given as mg/m²/day. § RT 50–50.4 Gy in 25–28 fractions.

© 2020 by the authors. Submitted for possible open access publication under the terms and conditions of the Creative Commons Attribution (CC BY) license (http://creativecommons.org/licenses/by/4.0/).
